# Factors associated with cancer worries in individuals participating in annual pancreatic cancer surveillance

**DOI:** 10.1007/s10689-016-9930-4

**Published:** 2016-09-14

**Authors:** Ingrid C.A.W. Konings, Femme Harinck, Marianne A. Kuenen, Grace N. Sidharta, Jacobien M. Kieffer, Cora M. Aalfs, Jan-Werner Poley, Ellen M.A. Smets, Anja Wagner, Anja van Rens, Frank P. Vleggaar, Margreet G.E.M. Ausems, Paul Fockens, Jeanin E. van Hooft, Marco J. Bruno, Eveline M.A. Bleiker, On behalf of the Dutch research group on pancreatic cancer surveillance in high-risk individuals

**Affiliations:** 1 Department of Gastroenterology and Hepatology, Erasmus MC, University Medical Center Rotterdam, Rotterdam, The Netherlands; 2 Division of Psychosocial Research and Epidemiology, The Netherlands Cancer Institute, Plesmanlaan 121, 1066 Amsterdam, The Netherlands; 3 Department of Clinical Genetics, Academic Medical Center, Amsterdam, The Netherlands; 4 Department of Medical Psychology, Academic Medical Center, Amsterdam, The Netherlands; 5 Department of Clinical Genetics, Erasmus MC, University Medical Center Rotterdam, Rotterdam, The Netherlands; 6 Family Cancer Clinic, The Netherlands Cancer Institute, Amsterdam, The Netherlands; 7 Department of Gastroenterology and Hepatology, University Medical Center Utrecht, Utrecht, The Netherlands; 8 Department of Clinical Genetics, University Medical Center Utrecht, Utrecht, The Netherlands; 9 Department of Gastroenterology and Hepatology, Academic Medical Center, Amsterdam, The Netherlands

**Keywords:** Cancer worries, Pancreatic cancer, Surveillance, High-risk individuals, Psychosocial burden, Predictive factors

## Abstract

It is important to adequately and timely identify individuals with cancer worries amongst participants in a pancreatic ductal adenocarcinoma (PDAC) surveillance program, because they could benefit from psychosocial support to decrease distress. Therefore, the aim of this study was to assess both psychosocial and clinical factors associated with cancer worries. High-risk individuals participating in PDAC-surveillance were invited to annually complete a cancer worry scale (CWS) questionnaire which was sent after counseling by the clinical geneticist (T0), after intake for participation in PDAC-surveillance (T1), and then annually after every MRI and endoscopic ultrasonography (EUS) (T2 and further). Analyses were performed to identify factors associated with cancer worries in the second year of surveillance (T3). We found a significant intra-individual decrease in cancer worries (β = −0.84, *P* < 0.001), nevertheless, 33 % of individuals had a CWS-score ≥14 at T3. We found one factor significantly associated with cancer worries at T3: having a family member affected by PDAC <50 years of age (β = 0.22, *P* = 0.03). The detection of a cystic lesion, a shortened surveillance interval, or undergoing pancreatic surgery did not lead to more cancer worries (*P* = 0.163, *P* = 0.33, and *P* = 0.53, respectively). In conclusion, this study identified ‘a family history of PDAC <50 years of age’ as the only predictor of cancer worries experienced after 2 years of surveillance in individuals at high risk of developing PDAC. This knowledge could help clinicians to timely identify individuals ‘at risk’ for high levels of cancer worries who would likely benefit from psychosocial support.

## Introduction

Pancreatic ductal adenocarcinoma (PDAC) is a deadly disease: despite its relatively low incidence of 10–12 new cases per 100,000 persons/year [1–3], PDAC is ranked among the top five causes of cancer-related deaths [4, 5]. Its 5-year survival rate has not significantly improved over the past decades and is less than 6 % [4, 5]. Since survival rates strongly depend on the stage of PDAC when detected, there is globally an increasing interest in surveillance to detect PDAC or its precursor high-grade dysplastic lesions at an early stage. Although screening of the entire population for PDAC is unlikely to be feasible because of the lack of a non-invasive, reliable and affordable surveillance tool, surveillance of well-defined high-risk groups for PDAC might be effective.

Two specific groups of individuals are considered to be at high risk of developing PDAC: (1) mutation carriers of hereditary syndromes that increase the risk of developing PDAC (i.e. carriers of mutations in the *CDKN2A*, *BRCA1, BRCA2* or *TP53* gene, and individuals with Peutz–Jeghers or Lynch syndrome), and (2) individuals without a known gene mutation but who have a strong family history of PDAC [familial pancreatic cancer (FPC)]. In these individuals, the risk of developing PDAC can be up to 75-fold higher than in the general population [6–13].

Over the past decades, multiple studies into the effectiveness of surveillance for PDAC in high-risk individuals have been performed [14–25]. Importantly, however, when assessing the effectiveness of a surveillance program, one should also take into account the psychological aspects of repeated participation in such a surveillance program. We previously reported that repeated participation in annual surveillance imposed low psychological burden on individuals at high risk for PDAC. However, we did find that a third of the participants had moderate to high cancer worries [26].

As individuals with high levels of cancer worries might benefit from psychosocial support to decrease the levels of psychological distress, it could be essential to adequately and timely identify these individuals. Therefore, the aim of this study was (1) to evaluate the course of cancer worries over a 2-year period of PDAC-surveillance (2) to identify psychosocial factors associated with cancer worries, and (3) to assess the impact of pancreatic cystic lesion detection, a recommended shortened surveillance interval, and undergoing pancreatic surgery on cancer worries in high-risk individuals participating in annual PDAC-surveillance.

## Methods

### Participants

All participants of an ongoing Dutch pancreatic cancer surveillance study (FPC-study) were invited to participate in a psychological questionnaire study as previously described [26]. The FPC-study is an ongoing multicenter prospective study investigating the effectiveness of PDAC-surveillance in high-risk individuals. Eligible for inclusion in this study are asymptomatic individuals with an estimated familial or hereditary life-time risk of developing PDAC ≥10 % (see inclusion criteria in Table 1). The minimal age for inclusion between 2008 and 2013 was 45 years of age (or 30 years in case of Peutz–Jeghers syndrome) or 10 years younger than the age of the youngest relative with PDAC, whichever age occurred first. Since 2013, the minimal age for inclusion is 50 or 10 years younger than the age of the youngest relative with PDAC. Surveillance ends at the age of 75. All potential candidates are evaluated by a clinical geneticist prior to inclusion. They are informed that the effectiveness of PDAC surveillance in reducing morbidity and mortality is not yet proven.

**Table 1:** Inclusion criteria for the pancreatic cancer surveillance study

Carriers of *CDKN2A* gene mutations, regardless of the family history of PDAC
Peutz–Jeghers syndrome patients (diagnosis based on a proven *LKB1/STK11* gene mutation or clinical signs), regardless of the family history of PDAC
Carriers of gene mutations in *BRCA1*, *BRCA2*, *TP53*, or Mismatch Repair genes with a family history of PDAC in ≥2 family members
Individuals with ≥2 relatives affected by pancreatic cancer who were related in the first degree to each other, of which at least one was related in the first-degree to the eligible individual
Individuals with ≥3 relatives affected by pancreatic cancer who were related in the first or second degree to each other, of which at least one was related in the first-degree to the eligible individual
Individuals with ≥2 relatives affected by pancreatic cancer who were related in the second degree to each other, of which at least one was related in the first-degree to the eligible individual and at least one was aged under 50 years at time of diagnosis

*PDAC* pancreatic ductal adenocarcinoma

### Clinical study procedures

The clinical study procedures were previously extensively described [25]. In summary, annual surveillance of the pancreas is performed using endoscopic ultrasonography (EUS), carried out by experienced endosonographers, and magnetic resonance imaging (MRI) with intravenous administration of gadobutrol. EUS is performed under conscious (midazolam/fentanyl) or propofol sedation. Some participants undergo surveillance with only MRI or EUS (see Table 2) due to contra-indications for either modality (for example claustrophobia, pacemaker or discomfort during initial EUS). Follow-up policy is based on the agreement of an expert panel consisting of endosonographists, surgeons, radiologists and pathologists and is as follows:

**Table 2:** Baseline characteristics of study participants

	All individuals (n = 166) N (%)	Individuals with the T0, T1 and/or T2 AND the T3 questionnaire (n = 117) N (%)	Individuals without the T0, T1 and/or T2 NOR the T3 questionnaire (n = 49) N (%)	*P* value (n = 117 vs. n = 49)
Age at inclusion, mean (range, SD)	51 (19–73, 9.7)	51 (19–73, 9.5)	51 (30–72, 10.3)	0.894
Gender, male	68 (41 %)	50 (43 %)	18 (37 %)	0.473
Genetic background				
Familial pancreatic cancer (FPC)	84 (51 %)	60 (51 %)	24 (49 %)	
CDKN2A (FAMMM syndrome)	44 (27 %)	32 (27 %)	12 (25 %)	
BRCA1 (HBOC)	2 (1 %)	2 (2 %)	0 (0 %)	
BRCA2 (HBOC)	25 (15 %)	17 (15 %)	8 (16 %)	
LKB1 (Peutz–Jeghers syndrome)	7 (4 %)	4 (3 %)	3 (6 %)	
TP53 (Li Fraumeni syndrome)	4 (2 %)	2 (2 %)	2 (4 %)	0.783
Number of PDAC cases in the family, mean (range, SD)	2 (0–7, 1.2)	2 (0–7, 1.2)	2 (0–5, 1.2)	0.202
Youngest family member affected by PDAC, mean (range, SD)	51 (21–89, 11.4)	51 (21–89, 11.4)	53 (40–80, 11.4)	0.357
Children				
Yes	136 (82 %)	104 (89 %)	32 (65 %)	
No	20 (12 %)	11 (9 %)	9 (18 %)	
No data	10 (6 %)	2 (2 %)	8 (16 %)	**0.042**
Marital status				
Married/co-habiting/LAT relationship	129 (78 %)	98 (84 %)	31 (63 %)	
Single/divorced/widowed	19 (11 %)	11 (9 %)	8 (16 %)	
No data	18 (11 %)	8 (7 %)	10 (20 %)	0.095
Level of education				
Primary school	3 (2 %)	3 (3 %)	0 (0 %)	
High school	39 (24 %)	27 (23 %)	12 (25 %)	
College/university	115 (69 %)	85 (73 %)	30 (61 %)	
No data	9 (5 %)	2 (2 %)	7 (14 %)	0.486
Smoking behavior				
Never smoker	85 (51 %)	60 (51 %)	25 (51 %)	
Current or past smoker	67 (40 %)	50 (43 %)	17 (35 %)	
No data	14 (8 %)	7 (6 %)	7 (14 %)	0.580
Alcohol consuming				
Never consumer	37 (22 %)	30 (26 %)	7 (14 %)	
Current or past consumer	114 (69 %)	81 (69 %)	33 (67 %)	
No data	15 (9 %)	6 (5 %)	9 (18 %)	0.230
Ever treated for cancer				
Any type of cancer	47 (28 %)	35 (30 %)	12 (25 %)	
Melanoma	28 (17 %)	20 (17 %)	8 (16 %)	
Breast cancer	13 (8 %)	10 (9 %)	3 (6 %)	
Other	10 (6 %)	9 (8 %)	1 (2 %)	0.479
Surveillance with				
EUS & MRI	159 (96 %)	112 (96 %)	47 (96 %)	
EUS only	2 (1 %)	2 (2 %)	0 (0 %)	
MRI only	5 (3 %)	3 (3 %)	2 (4 %)	0.576

*SD* standard deviation, *FAMMM* familial atypical multiple mole melanoma, *HBOC* hereditary breast and ovarian cancer, *PDAC* pancreatic ductal adenocarcinoma, *LAT* living apart together, *EUS* endoscopic ultrasonography, *MRI* magnetic resonance imaging

Bold *P*-values are considered statistically significant

1. Annual surveillance when either no pancreatic abnormalities or cystic lesions <10 mm are detected;
2. Interval surveillance after 6 months when a novel cystic lesion is detected with a diameter of 10–30 mm without worrisome features;
3. Interval surveillance after 3 months when a lesion of unknown significance is detected for which there is no unanimous opinion amongst members of the expert panel;
4. Surgical resection in case of 1. a solid lesion which is considered suspicious for malignancy, 2. a cystic lesion ≥30 mm, 3. a cystic lesion with worrisome features (thickened/enhanced cyst wall and/or mural nodules), or 4. a main branch intraductal papillary mucinous neoplasm (IPMN, main pancreatic duct ≥10 mm).

### Questionnaire study

**Fig. 1:**
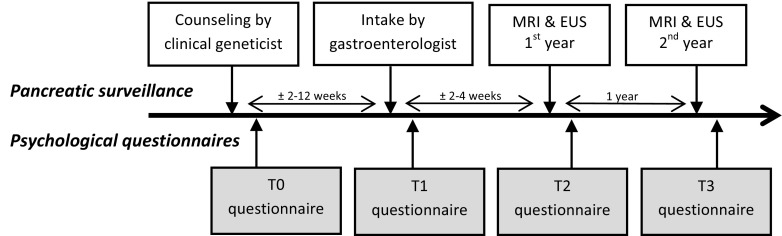
Overview of both the clinical part of the pancreatic cancer surveillance study and the timing of the psychological questionnaires. *MRI* magnetic resonance imaging, *EUS* endoscopic ultrasonography

All participants of the ongoing PDAC-surveillance study are invited to participate in the ongoing prospective multicenter psychological questionnaire study. Participants receive a first questionnaire on sociodemographic data after their counseling session with the clinical geneticist (T0), a second questionnaire after explanation of the study procedures by the gastroenterologist (T1), and then annually after receiving their surveillance results (T2 and further), see also Fig. 1. Because this questionnaire study was added after the first inclusion period of the original clinical study protocol, some participants had already had their first investigations and therefore started their questionnaires at T2.

All measurements used in the questionnaires were previously described [26]. We report here the results of the cancer-related worries as assessed with the eight-item cancer worry scale (CWS) [27, 28]. The items of the CWS are shown in Table 3. The total CWS-score ranges from 8 to 32, with higher scores indicating more frequent worries about cancer. There is no clear cut-off point for the CWS-score, nevertheless, a score ≥14 could be indicative of moderate to high levels of cancer worries [29]. The Cronbach’s alpha, a measure of internal consistency with values >0.70 being considered acceptable, was high for the CWS in the current sample at T3 (0.86, n = 121).

The ethical committee of all participating centers approved the study protocol and the study was conducted in accordance with the declaration of Helsinki. All participants gave written informed consent prior to the performance of any study-related investigations.

### Statistical analyses

Questionnaires were analyzed using descriptive statistics. Intra-individual change in cancer worries over time was assessed with a mixed-effect model (growth curve model) with a maximum likelihood estimator and unstructured covariance matrix. Univariate and multivariate regression analyses were performed to identify sociodemographic factors from the questionnaires T0, T1 and/or T2 that were associated with cancer worries at the second year of follow-up (T3). For these analyses, we selected all participants who returned the T3 questionnaire as well as at least a T0, T1 or T2 questionnaire. To analyze the impact on cancer worries of the detection of a pancreatic cystic lesion, a recommended shortened surveillance interval, and undergoing pancreatic surgery, we selected all participants who returned the questionnaire in the year of the event (i.e. the detection of a cyst and/or an advised shortened surveillance interval and/or undergoing pancreatic surgery; the questionnaire was sent after participants had received their surveillance results) and who returned the questionnaire 1 year before and/or 1 year after the event. A paired-samples *T* test was performed for these analyses. In all analyses, a *P* value <0.05 was considered statistically significant. All analyses were conducted using the statistical package for the social sciences (version 21, SPSS Institute, Chicago, IL).

## Results

### Participants’ characteristics

In March 2015, 166 individuals participated in the questionnaire study. Baseline characteristics of all individuals are summarized in Table 2. Mean age of all 166 participants at inclusion in the clinical study was 51 years, of whom 47 (28 %) were treated for cancer (predominantly for melanoma or breast cancer) prior to inclusion in the study.

### Cancer worries

The scores per item on the CWS-questionnaires are shown in Table 3. The mean CWS-score was 14 at T0, 14 at T1, 13 at T2, and 12 at T3; the overall average CWS-score was 13. We found a significant intra-individual decrease in the CWS-score over time (β = −0.84, *P* < 0.001). Thirty-nine individuals (33 %) had a CWS-score ≥14 in the second year of follow-up (T3), this was 51, 52 and 43 % at T0, T1 and T2, respectively.

**Table 3:** Scores on the CWS-questionnaire, shown per item per questionnaire

Item During the last 7 days	T0 *n* = 36 % often/always worried	T1 *n* = 80 % often/always worried	T2 *n* = 148 % often/always worried	T3 *n* = 121 % often/always worried	Average (on T0–T3) % often/always worried
How often have you thought about your chances of getting cancer (again)?	19	13	10	5	10
Have these thoughts affected your mood?	11	5	2	4	4
Have these thoughts interfered with your ability to do daily activities?	0	4	1	1	1
How concerned are you about the possibility of getting cancer one day?	33	26	26	19	25
How often do you worry about developing cancer?	25	11	13	7	12
How much of a problem is this worry?	11	6	5	3	5
How often do you worry about the chance of family members developing cancer?	28	25	20	12	20
How concerned are you about the possibility that you will ever need surgery (again)?	14	13	8	5	9
Mean CWS-score (range, SD)	14.4 (8–26, 4.3)	13.9 (8–26, 3.8)	13.3 (8–25, 3.4)	12.2 (8–25, 3.3)	13.2* (8–26, 3.6)

*CWS* cancer worry scale, *SD* standard deviation

* Significant (β = −0.84, *P* < 0.001) intra-individual decrease over time [in comparison with first assessment (T0)], non-proportional analysis

### Factors associated with cancer worries at the second year of follow-up

For these sub-analyses, we only included individuals with a T3 assessment, as well as at least a T0, T1 or T2 assessment. Of the 166 individuals that participated in the questionnaire study, 117 individuals returned the T3 questionnaire as well as at least a T0, T1 and/or T2 questionnaire (response 70 %). Baseline characteristics for these 117 individuals selected for sub-analyses, and for the 49 individuals without the required questionnaires, are summarized in Table 2. The subgroup of 117 individuals only differed in comparison to the excluded individuals (n = 49) on having children (89 % of the included individuals had children vs. 65 % of excluded individuals, *P* = 0.04).

**Fig. 2:**
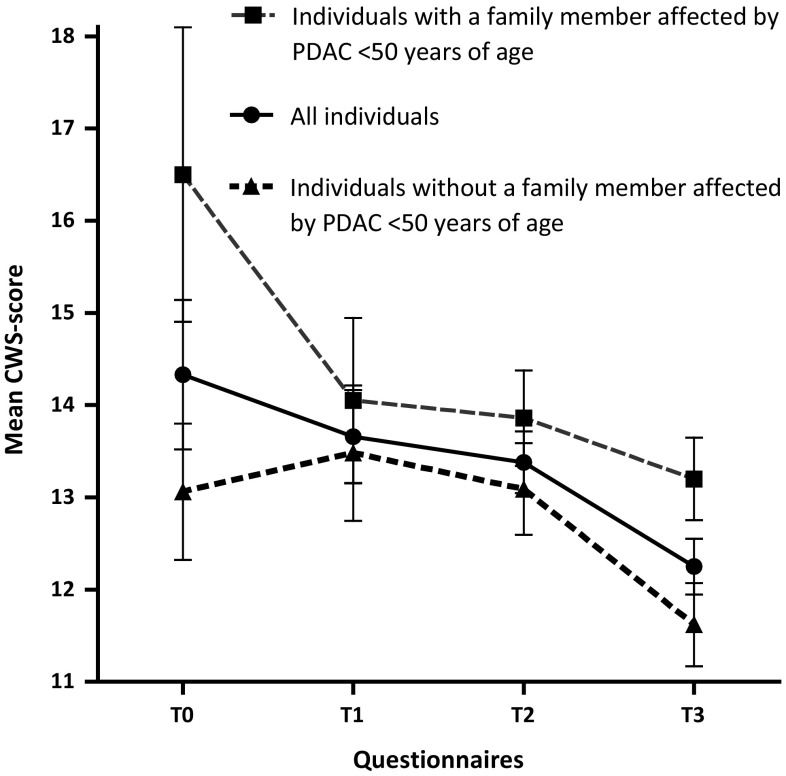
Mean CWS-scores at different moments in time, shown for all individuals and for individuals with and without a family member that was affected by pancreatic cancer under 50 years of age *CWS* cancer worry scale, *PDAC* pancreatic ductal adenocarcinoma

For the selection of possible predictors of cancer worries in the second year of follow-up (T3), we performed univariate regression analyses. Significant predictors were ‘having a family member affected by PDAC below the age of 50′ (β = 0.23, *P* = 0.01), and ‘a perceived elevated risk of developing PDAC’ (β = 0.23, *P* = 0.01). Not predictive were, amongst other factors, the number of PDAC-cases in the family and a personal history of cancer, see also Table 4. In the next step, the two significant predictors were included in the multivariate model, together with age, gender and genetic background. In this multivariate analysis (see Table 4), having a family member affected by PDAC below the age of 50 was associated with cancer worries in the second year of follow-up (β = 0.22, *P* = 0.03). Figure 2 shows the mean CWS-score per questionnaire for all individuals and for individuals with and without a family member affected by PDAC <50 years of age.

**Table 4:** Univariate and multivariate analysis for factors possibly associated with cancer worries in the second year of follow-up (T3)

Factors	N (%)/mean (range, SD)	Univariate analyses		Multivariate analysis	
		β	*P* value	β	*P* value
Age at inclusion, mean (range, SD)	51 (19–73, 9.5)	−0.142	0.126	0.010	0.924
Female gender	67 (57 %)	0.140	0.133	0.119	0.215
Carriership of a gene mutation	57 (49 %)	0.172	0.063	0.133	0.183
Number of PDAC cases in the family, mean (range, SD)	2 (0–7, 1.2)	0.058	0.538		
Having a family member affected by PDAC <50 years of age	45 (39 %)	0.234	**0.016**	0.218	**0.031**
Having children	104 (89 %)	0.033	0.723		
Being in a relationship	98 (84 %)	−0.046	0.635		
Education at college/university-level	85 (73 %)	−0.001	0.995		
Current or past smoker	50 (43 %)	0.140	0.143		
Current or past alcohol consumer	81 (69 %)	−0.031	0.744		
Personal history of any type of cancer	35 (30 %)	0.048	0.610		
Body Mass Index, mean (range, SD)	25.8 (10.0–43.8, 4.6)	0.085	0.233		
Perception of moderately to strongly elevated risk of developing PDAC	69 (59 %)	0.228	**0.013**	0.163	0.109
Previous psychological support	17 (15 %)	0.181	0.053		
Having someone available to confide in	111 (95 %)	−0.077	0.407		

*SD* standard deviation, *PDAC* pancreatic ductal adenocarcinoma

Bold *P*-values are considered statistically significant

### Impact of the detection of a pancreatic cystic lesion on cancer worries

In 93 out of all the 166 participants (56 %), a pancreatic cystic lesion was detected during surveillance. Forty of these 93 individuals (43 %) returned the questionnaire the year prior to the detection of the cystic lesion (mean CWS-score 13.3, standard deviation (SD) 3.6), as well as the questionnaire in the year of the detection of the lesion (mean CWS-score 12.5, SD 3.7). The difference in mean CWS-score was not statistically significant (95 % CI for the difference −0.3 to 1.9, *P* = 0.163). A total of 45 individuals (48 %) returned the questionnaire in the year of detection (mean CWS-score 11.9, SD 3.5) as well as the questionnaire 1 year after detection (mean CWS-score 11.9, SD 3.4). Again, the difference in mean CWS-score between the 2 years was not statistically significant (95 % CI for the difference −1.1 to 1.1, *P* = 0.97).

### Impact of a recommended shortened surveillance interval on cancer worries

For 25 out of 166 individuals (15 %), a shortened surveillance interval was recommended; for 16 individuals an interval of 3 months and for nine individuals an interval of 6 months. Six of these 25 individuals (24 %) returned the questionnaire in the year prior to the shortened surveillance interval (mean CWS-score 14.3, SD 3.8), as well as in the year of the shortened surveillance interval (mean CWS-score 15.5, SD 4.7). The difference in mean CWS-score of 1.2 points was not significant (95 % CI for the difference −3.9 to 1.6, *P* = 0.33). Nine individuals (36 %) returned the questionnaire in the year of the shortened surveillance interval (mean CWS-score 14.4, SD 5.2), as well as in the year after (mean CWS-score 12.2, SD 4.5). This decrease in mean CWS-score by 2.2 points was also not statistically significant (95 % CI for the difference −1.0 to 5.4, *P* = 0.15).

### Impact of pancreatic surgery on cancer worries

In 7 out of 166 individuals (4 %), pancreatic surgery was performed. Two of these individuals returned both the questionnaire from the year prior to surgery (mean CWS-score 10.5 (SD 3.5), as well as the post-operative questionnaire in the year of surgery (mean CWS-score 11.0, SD 0.0). The difference in mean CWS-score was not statistically significant (*P* = 0.87). Four cases returned both the questionnaire in the year of surgery (mean CWS-score 14.0, SD 3.5), as well as the questionnaire in the year after surgery (mean CWS-score 11.8, SD 3.9). This decrease in score by 2.2 points was not statistically significant (95 % CI for the difference −7.9 to 12.4, *P* = 0.53).

## Discussion

In this prospective multicenter study, we assessed the course of cancer worries over a 2-year period in high-risk individuals participating in annual PDAC-surveillance, assessed demographic baseline and psychosocial factors that could be associated with these cancer worries, as well as the impact of three clinical events on cancer worries. Independently associated with cancer worries in the second year of follow-up was having a family member that was affected by PDAC below the age of 50.

Because PDAC-surveillance is being performed more and more worldwide, it is key to take into account the psychological aspects of repeated participation. Although we previously reported a low general psychological burden of annual participation in PDAC-surveillance [27], 33 % of participants did have cancer-specific worries with a CWS-score ≥14. While this is not a rigorously tested cut-off point and there are no norm-data on cancer worries in the general population, a score ≥14 is considered to be indicative of moderate to high cancer worries [29]. It is important to adequately and timely identify these individuals with cancer worries, because they would likely benefit from psychosocial support to decrease or prevent psychological distress. Psychosocial interventions, varying from psycho-education and mindfulness-training to cognitive behavioral therapy, have been proven to be effective in reducing levels of distress to such levels that patients can resume their daily activities.

Therefore, this study focused on cancer worries during PDAC-surveillance, more specifically on the course of cancer worries over time, on predictors of cancer worries, and on cancer worries during certain events. To our current knowledge, this is the first study with a prospective design assessing these characteristics of cancer worries in individuals at inherited or familial high risk of developing PDAC over time. Although much research was done into generalized distress and levels of cancer worries, factors influencing cancer worries were hardly studied in populations at inherited high risk of developing other types of cancer [27, 30–34]. Sociodemographic and clinical variables found to be significantly associated with cancer-specific distress for familial adenomatous polyposis (FAP) were lower educational level, female gender, diagnosis of FAP (as opposed to being at risk for FAP or being a non-carrier), having a personal history of cancer, and having had surgery more than 10 years ago [27]. In individuals with Lynch syndrome, however, no difference for age, gender, level of education, actual or perceived risk of Lynch syndrome, or a personal history of cancer was found [30]. In a Von Hippel–Lindau (VHL) population, factors associated with VHL-related worries were diagnosis of, or treatment for, VHL, a high level of social constraint, a high perceived risk of developing tumors, and the loss of a close relative due to VHL during adolescence [31].

As in our previous study [27], individual cancer worries decreased over the 2-year period of surveillance in high-risk individuals for PDAC. We identified a perceived elevated risk of developing PDAC and having a family member that was affected by PDAC under 50 years of age as factors associated with cancer worries in the second year of follow-up, the latter being independently associated. Both factors resemble the findings by Lammens et al. [31], who described a high perceived risk of developing tumors and the loss of a close relative during adolescence as related to cancer-specific worries.

Surprisingly, a factor not associated with high cancer worries, was a personal history of cancer. This factor was previously described as associated with high cancer worries [27], and one might expect individuals who already had cancer in the past to be more anxious of developing cancer again, especially when being at high risk of this. Educational level was also not associated with high cancer worries at the second year of follow-up, in contrast to a previous study in FAP-individuals [27].

We also assessed three clinical events for association with increased cancer worries: the detection of a cystic lesion, a recommended shortened surveillance interval, and undergoing pancreatic surgery. For all three events, we did not find a significant change in CWS-score for the year prior to the event and/or the year after the event in comparison to the year of the event. However, the CWS-score in participants with a recommended shortened surveillance interval did differ considerably between that year and the year after the event, and so did the CWS-score in the individuals who underwent surgery. This suggests that a shortened surveillance interval and pancreatic surgery cause a decrease in CWS-score the year after, possibly due to relief at follow-up, however, our sample size for these sub-analyses (n = 9 and n = 4) were likely too small to find a statistically significant difference, which is also demonstrated by the large 95 % confidence interval for the differences in CWS-scores.

This study has several strengths. The prospective design in a large group of individuals at high risk of developing pancreatic cancer is unique and of great scientific value. However, this study also has some limitations, one of which might be the power for our sub-analyses on clinical factors. Therefore, to draw definite conclusions on these factors, a larger study sample is needed. Also, because the questionnaire study was added after the first inclusion period of the original clinical study protocol, some participants had already had their first investigations and therefore started their questionnaires at T2, which resulted in a relatively low number of available T0 questionnaires in the analyzed cohort.

In conclusion, this prospective questionnaire study identified the factor ‘having a family member affected by PDAC <50 years of age’ to be associated with cancer worries in the second year of follow-up in individuals at inherited or familial high risk of developing PDAC who are participating in annual surveillance. Recognizing this factor can help clinicians to timely identify individuals ‘at risk’ of a high level of cancer worries whom would likely benefit from psychosocial support to decrease or prevent psychological distress.
